# Surgical castration versus chemical castration in donkeys: response of stress, lipid profile and redox potential biomarkers

**DOI:** 10.1186/s12917-020-02530-0

**Published:** 2020-08-26

**Authors:** Nasser S. Abou-Khalil, Marwa F. Ali, Magda M. Ali, Ahmed Ibrahim

**Affiliations:** 1 Department of Medical Physiology, Faculty of Medicine, Assiut University, Assiut, 71526 Egypt; 2 Department of Veterinary Pathology and Clinical Pathology, Faculty of Veterinary Medicine, Assiut University, Assiut, 71526 Egypt; 3 Department of Surgery, Anesthesiology and Radiology, Faculty of Veterinary Medicine, Assiut University, Assiut, 71526 Egypt; 4 Veterinary Teaching Hospital, Faculty of Veterinary Medicine, Assiut University, Assiut, 71526 Egypt

**Keywords:** Surgical castration, Chemical castration, Physiology, Stress, Lipid profile, Oxidative stress

## Abstract

**Background:**

Castration is a husbandry practice raising important questions on the welfare and physiological status of farm animals. Searching for effective castration methods that minimally compromise the body physiology is worthy of attention. Therefore, this study aimed to evaluate the differential response of biological systems in donkeys to surgical castration versus the chemical one by CaCl_2_ with special emphasis on stress, lipid profile, and oxidative stress biomarkers. Donkeys were divided randomly and equally into two groups; the chemical (Ch) and surgical (S) groups (*n* = 6). The Ch group was chemically castrated by intratesticular injection of 20% CaCl_2_ dissolved in absolute ethanol. Blood samples were collected prior to castration and at 15, 30, 45, and 60 days after the beginning of experiment.

**Results:**

Surprisingly, the Ch group at the end of the experiment was characterized by significantly higher cortisol level compared to the S group. TC and LDL-C levels in the S group significantly decreased at day 45, while TG levels significantly increased at days 45 and 60 in comparison with day 0. HDL-C levels at days 30 and 60 in the Ch group significantly increased in comparison with day 0. At day 30 post-castration, HDL-C was significantly higher and LDL-C was significantly lower in the Ch group than the S group. A significant elevation in TC and LDL-C was observed at day 45 and in HDL-C at the end of experimental duration in the Ch group when compared with the S group. TPX level was significantly lower and TAC was significantly higher in the Ch group at day 45 than the S group.

**Conclusion:**

Surgical castration evoked less stress and minor changes in lipid profile and oxidant/antioxidant balance relative to chemical castration by intratesticular 20% CaCl_2_ dissolved in absolute ethanol.

## Background

Our previous study revealed that a single bilateral intratesticular injection of 20% calcium chloride (CaCl_2_) dissolved in absolute ethanol in donkeys was ineffective in reducing serum testosterone levels over 60 days post-castration in contrast to the surgical castration using the scrotal ablation technique [1]. In continuation of this work, we decided to examine the difference in the effects of these two types of castration on some physiologically relevant parameters.

Although CaCl_2_ castration seems to be an appropriate non-surgical strategy in the terms of efficacy, cost, animal welfare and metabolic issues in albino rats, cats, dogs, and Black Bengal goats [2–7], little is known about its impacts on the physiological status of body across a time-window study. In an attempt to explore the dose-dependent response of different animal species to intratesticular injection of CaCl_2_, previous studies reported that this method of chemo-castration was not accompanied with long-term stress reactivity [3–6].

Whether the difference in the type of castration caused a fingerprint difference in modulating stress, lipid profile, and redox biomarkers had not been thoroughly examined. Regarding the effects of castration on lipoproteins, a broad spectrum of conflicting data has been reported in the literature. Orchiectomized pigs and cattle were characterized with up-regulated lipogenesis [8, 9], on the other hand castration failed to change plasma total cholesterol, high density lipoprotein-cholesterol, glycerol, and triglycerides levels in Sprague-Dawley rats [10]. Monitoring the temporal changes in plasma lipoproteins following castration may provide a better understanding their metabolic regulation under conditions of androgen deprivation. Disturbance in lipid metabolism evokes adverse consequences such as insulin resistance, hypertension, inflammation, and endothelial dysfunction [11–14].

Surgical castration resulted in oxidative stress in the blood of Wistar/ST rats and mixed-breed dogs [15, 16], also CaCl_2_ castration caused peroxidative injury in the testicular tissues of albino rats, dogs, and black Bengal goats [3–6]. These discrepancies add fuel to our study in the light of species-related differential response. Resulting from the imbalance between free oxygen/nitrogen species and free radical scavenging molecules, oxidative stress is predisposed to the development of bacterial, viral, and parasitic diseases thus, augmenting the prevalence of malignancies and autoimmune disorders [16].

Neural inputs from nociceptors together with unpleasant sensory and emotional experiences associated with tissue damage during castration activates the hypothalamic-pituitary-adrenal axis (HPA) leading to increased release of cortisol [17] initiating long-lasting metabolic and anti-inflammatory responses that can promote healing [18]. Cortisol is a reliable endocrinological biomarker of stress and provides indication of how unpleasant the experience is emotionally and physically as there is a direct association between the level of HPA activity and the degree of noxiousness [18]. Stressful conditions like castration cause imbalance between oxidants and antioxidants in favor of oxidants at the cellular and individual levels creating status of oxidative stress [19]. The greater the oxidative stress, the more severe the cellular damage would be during the surgery which may cause poor post-operation outcomes [15].

Alterations in lipid profile is considered as a valid indicator of stress [20, 21]. For instance, hypocholesterolemia is an obvious metabolic sign for exposure to stressful situations as cholesterol requirement increases to support the biosynthesis of stress hormones and the production and functions of new cells participate in host defense and tissue repair [22]. Stress hormones allow liberation of energy substrates through acting on adipose tissue metabolism during times of stress to supply the increased energetic needs of the body. They cause increase in the hydrolysis of triglycerides, the amount of fatty acids in circulation, and the de novo lipid production in hepatocytes [23].

The objective of this study was to examine the stress, lipid profile and redox responses of donkeys to standard surgical castration versus chemical castration using intratesticular injection of CaCl_2_ dissolved in absolute ethanol. We hypothesized that application of CaCl_2_, as a welfare-friendly castration method, is expected to induce less stress and less obvious changes in lipid metabolism and oxidant/antioxidant balance than the surgical castration according to the time-dependent changes in testosterone secretion in our previous work.

## Results

**Fig. 1:**
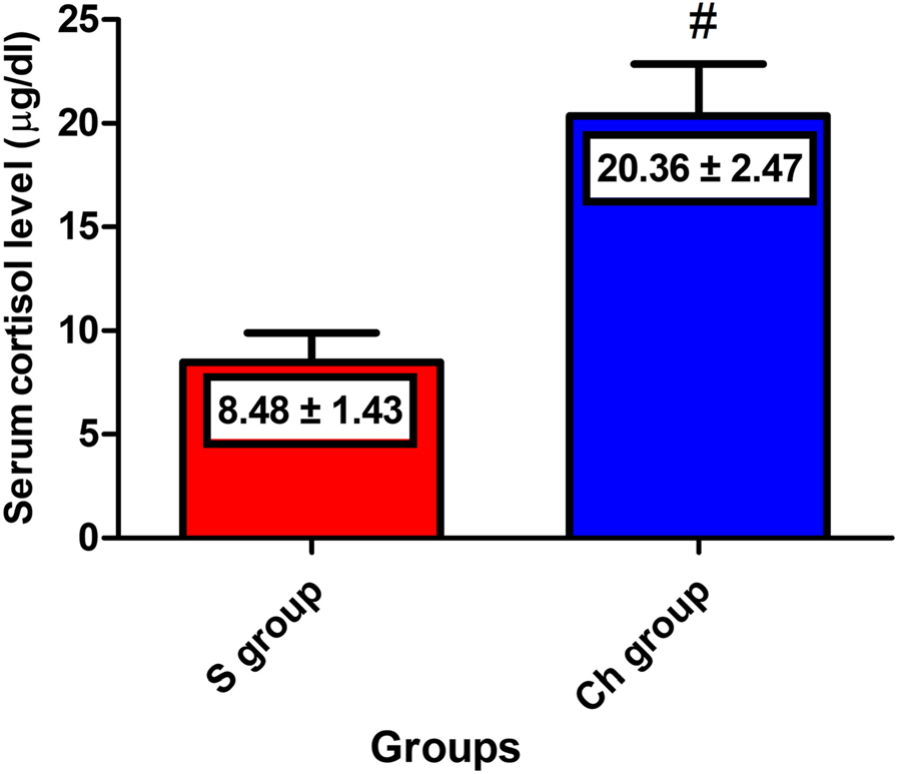
Serum cortisol concentrations in surgical castration (S) and chemical castration (Ch) groups at day 60 post-castration. Values are expressed as means ± SEM, *n* = 6 animals per group. ^#^ indicates significant difference between the two types of castration at day 60 post-castration using independent-samples t-test

As shown in Fig. 1, chemically castrated donkeys at the end of the experiment were characterized by significantly higher cortisol levels compared to the surgically castrated ones (20.36 ± 2.47 and 8.482 ± 1.43 μg/dl, respectively).

**Fig. 2:**
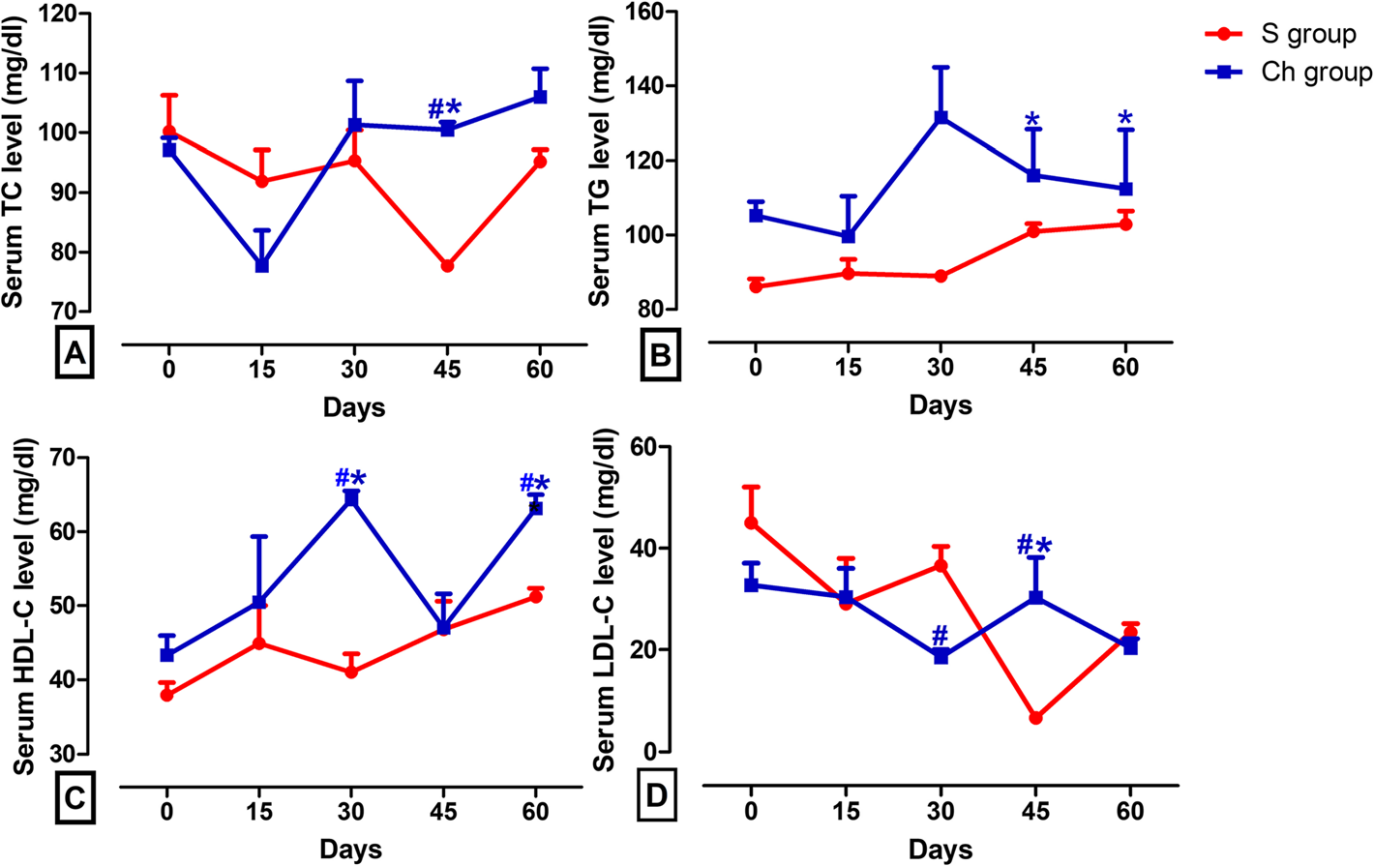
Serum concentrations of lipid profile in surgical castration (S) and chemical castration (Ch) groups. Graphic representation of changes in serum lipid profile levels of donkeys at days 15, 30, 45, and 60 following surgical or CaCl_2_ castration vs. day 0 (pre-castration). Values are expressed as means ± SEM, *n* = 6 animals per group. ^*^ indicates significant difference between each time point post-castration and day 0 using repeated measures ANOVA followed by Tukey’s multiple comparison test. ^#^ indicates significant difference between the two types of castration at each post-castration time point using independent-samples t-test. TC: total cholesterol; TG: triglycerides; HDL-C: high density lipoprotein-cholesterol; LDL-C: low density lipoprotein-cholesterol

When the surgical castration was used as a standard reference castration method, TC and LDL-C levels significantly decreased at day 45 (77.70 ± 0.35 and 6.60 ± 0.12 mg/dl, respectively), while TG levels significantly increased at day 45 and 60 (101.00 ± 2.08 and 102.90 ± 3.58 mg/dl, respectively) in comparison with day 0 (86.13 ± 2.06 mg/dl). However, there were no significant differences in HDL-C levels in the S group across the time window relative to the baseline level. Lack of significant difference was obvious in the S group when comparing pre-castration TC, LDL-C, and TG levels to the post-castration ones across all time intervals. HDL-C levels at day 30 and 60 (64.37 ± 1.17 and 63.17 ± 1.83 mg/dl, respectively) significantly increased in the chemically castrated group in comparison with day 0 (43.33 ± 2.60 mg/dl). At day 30 post-castration, HDL-C was significantly higher and LDL-C was significantly lower in the Ch group (64.37 ± 1.17 and 18.50 ± 1.52 mg/dl, respectively) compared to the S group (41.00 ± 2.52 and 36.57 ± 3.81 mg/dl, respectively). When comparing the Ch group to the S group, a significant elevation in TC (100.50 ± 1.28 and 77.70 ± 0.35 mg/dl, respectively) and LDL-C (30.31 ± 7.87 and 6.60 ± 0.12 mg/dl, respectively) was observed at day 45 and in HDL-C (63.17 ± 1.83 and 51.20 ± 1.15 mg/dl, respectively) at the end of the studied time frame (Fig. 2a-d, Table 1).

**Table 1:** Serum concentrations of lipid profile in the surgical castration (S) and chemical castration (Ch) groups

Time interval	0 day	15 day	30 day	45 day	60 day
Parameter					
***TC level (mg/dl)***					
**S**	100.20 ± 6.07	91.90 ± 5.24	95.37 ± 5.07	77.70 ± 0.35	95.20 ± 1.98
**Ch**	97.13 ± 2.03	77.73 ± 5.96	101.30 ± 7.36	100.50 ± 1.28^#^*	106.00 ± 4.70
***TG level (mg/dl)***					
**S**	86.13 ± 2.06	89.63 ± 3.88	89.00 ± 0.93	101.00 ± 2.08	102.90 ± 3.58
**Ch**	105.30 ± 3.67	99.63 ± 10.84	131.50 ± 13.53	116.10 ± 12.31^*^	112.50 ± 15.72^*^
***HDL-C level (mg/dl)***					
**S**	37.90 ± 1.70	44.90 ± 5.10	41.00 ± 2.52	46.80 ± 3.80	51.20 ± 1.15
**Ch**	43.33 ± 2.60	50.50 ± 8.84	64.37 ± 1.17^#*^	47.00 ± 4.62	63.17 ± 1.83^#*^
***LDL-C level (mg/dl)***					
**S**	45.04 ± 7.03	29.07 ± 8.98	36.57 ± 3.81	6.60 ± 0.12	23.43 ± 1.73
**Ch**	32.73 ± 4.33	30.42 ± 5.60	18.50 ± 1.52^#^	30.31 ± 7.87^#*^	20.37 ± 1.84

*TC* total cholesterol, *TG* triglycerides, *HDL-C* high density lipoprotein-cholesterol, *LDL-C* low density lipoprotein-cholesterol

**Fig. 3:**
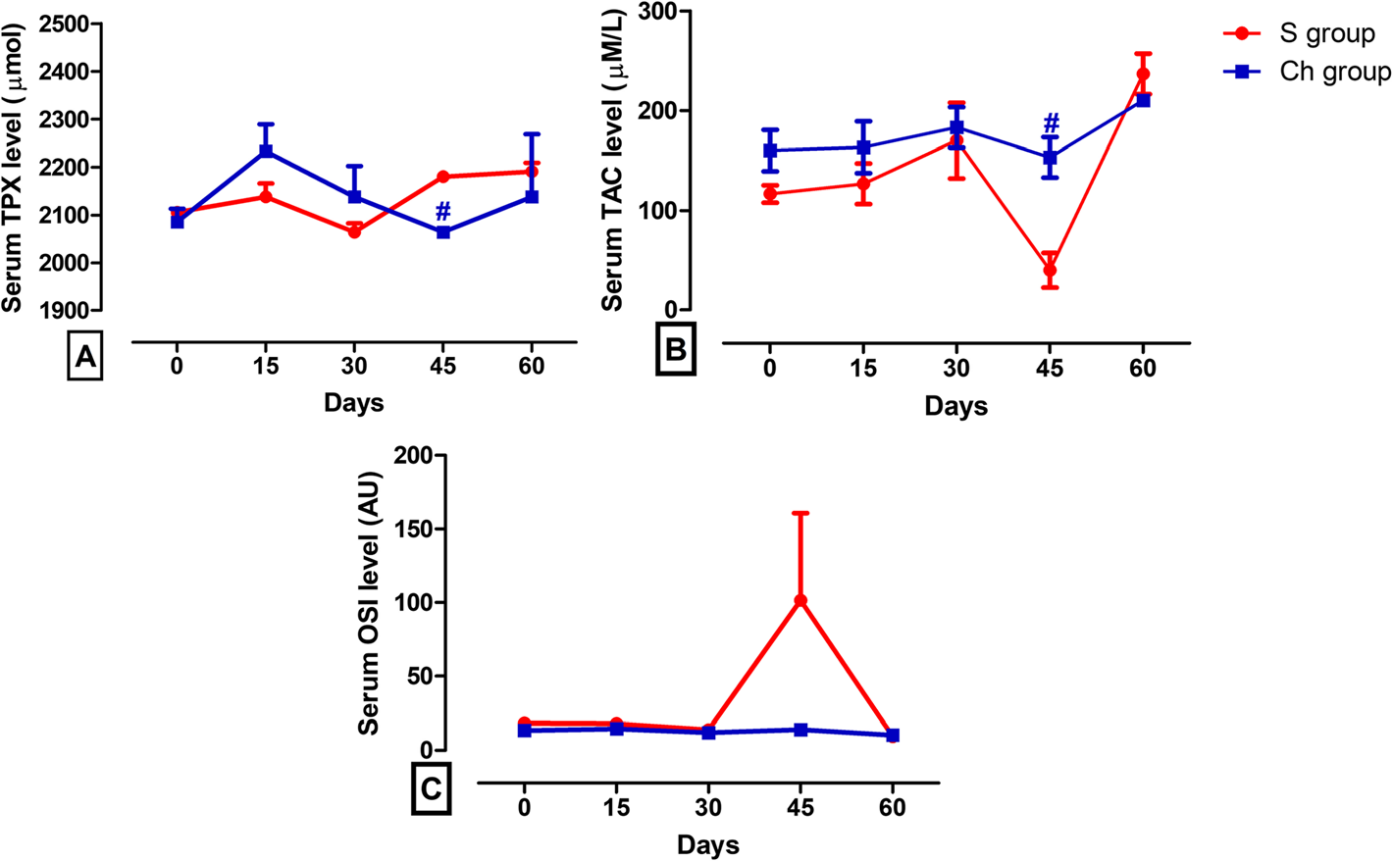
Serum concentrations of oxidant/antioxidant parameters in surgical castration (S) and chemical castration (Ch) groups. Graphic representation of changes in serum oxidant/antioxidant levels of donkeys at days 15, 30, 45, and 60 following surgical or CaCl_2_ castration vs. day 0 (pre-castration). Values are expressed as means ± SEM, *n* = 6 animals per group. ^*^ indicates significant difference between each time point post-castration and day 0 using repeated measures ANOVA followed by Tukey’s multiple comparison test. ^#^ indicates significant difference between the two types of castration at each post-castration time point using independent-samples t-test. TPX: total peroxide; TAC: total antioxidant capacity; OSI: oxidative stress index; AU: arbitrary unit

Figure 3a-c), Table 2 revealed the absence of significant difference between the post-castration TAC, TPX and OSI versus the pre-castration ones in both the Ch group and S groups. However, TPX level was significantly lower and TAC was significantly higher in the chemically castrated group at day 45 (2064 ± 0.00 μmol and 153.30 ± 20.28 μM/L, respectively) in comparison with the surgically castrated one (2180 ± 10.57 μmol and 40.00 ± 17.32 μM/L, respectively).

**Table 2:** Serum concentrations of oxidant/antioxidant parameters in the surgical castration (S) and chemical castration (Ch) groups

Time interval	0 day	15 day	30 day	45 day	60 day
Parameter					
***TPX level (μmol)***					
**S**	2106 ± 10.57	2138 ± 27.97	2064 ± 18.31	2180 ± 10.57	2191 ± 18.31
**Ch**	2085 ± 27.97	2233 ± 55.95	2138 ± 64.31	2064 ± 0.00^#^	2138 ± 129.9
***TAC level (***μm***/L)***					
**S**	116.70 ± 8.82	126.70 ± 20.28	170.00 ± 37.86	40.00 ± 17.32	236.70 ± 20.28
**Ch**	160.00 ± 20.82	163.30 ± 26.03	183.30 ± 20.28	153.30 ± 20.28^#^	210.00 ± 5.77
***OSI (AU)***					
**S**	18.28 ± 1.54	17.78 ± 2.86	13.71 ± 3.56	101.60 ± 59.16	9.39 ± 0.75
**Ch**	13.44 ± 1.58	14.51 ± 2.66	12.03 ± 1.67	13.94 ± 1.83	10.22 ± 0.85

*TPX* total peroxide, *TAC* total antioxidant capacity, *OSI* oxidative stress index, *AU* arbitrary unit

## Discussion

In contrast to our hypothesis, CaCl_2_ chemo-castration induced more distress than the surgical castration and mostly increased both of pro-atherogenic and anti-atherogenic lipoproteins. On the other hand, the surgical castration caused only a transient oxidative stress, which was overcame by enhancement in the body antioxidant capacity.

The animals received Different drug regimens at different procedures, which were unlikely to cause an effect at 15 days, where the first sample of blood was obtained, because of their short withdrawal time (4–5 days) [24]. The marked increase in serum cortisol levels in the chemically castrated donkeys versus the surgically castrated ones represents a major surprise. In the light of earlier studies [3–7], CaCl_2_ castration in rats, cats, dogs, or goats did not cause changes in a wide array of stress biomarkers including cortisol, corticosterone, fasting blood glucose, blood urea nitrogen, total plasma protein, rectal temperature and packed cell volume. However, orchiectomy by chemical substances like hypertonic sodium chloride was associated with more stress responses in the short term than the surgical methods in cattle. Acute cortisol level increased at 30 and 60 min following the procedure, but the increased levels in the chemically castrated group found only at 30 min. Nevertheless, when chronic discomfort biomarkers including cortisol and paraoxonase 1 were measured daily following the intervention up to 4 days, no significant differences were observed [25]. In a previous study, intratesticular injection of 30 (w/v) CaCl_2_ solution in water buffalo induced higher amount of acute phase protein (fibrinogen), as a stress indicator, than castration by Burdizzo clamp [26].

Stressful situations, including castration, activate HPA resulting in augmented cortisol secretion, making it a valid stress bio-indicator [27, 28]. Nevertheless, the presence of nociceptors exclusively in the testicular capsule and scrotal skin rather than the testicular and epididymal parenchyma makes the pain sensation occur as a result of increased intratesticular pressure during and immediately after injection [7, 29], and weakens the possibility of its occurrence on the long term after injection. Although monitoring change in the cortisol levels is used most frequently to assess the painful stress induced by castration, this biomarker could be confounded by factors other than pain and require frequent sampling to capture transient or pulsatile changes [30]. Because no optimal measure of stress exists, the utilization of multiple different measures such as changes in behavioral patterns, and measurement of eye temperature and locomotor activity [25, 31, 32] are highly warranted to get a full dimensional picture and reinforce and facilitate interpretation of findings of the current study. On the other hand, pain assessment methods have several limitations in equine [33]. This is because the difficulty in quantifying pain in this species [34], and that the pain-related behaviours do not specify the source and type of pain, nor do they correlate strictly with severity of pain or progression of a disorder [35]. Even composite pain scale, which involves scoring of both behavioural and physiological variables, has limited utility in discriminating mild pain [36].

In the current study, signs of overt pain were absent in donkeys post-operatively. This is may be due to the use of successful anesthetic protocols, followed by the regular administration of pain medications. Also, absence of obvious pain signs could be attributed to the fact that donkey has higher tolerance to painful stimuli [35]. There is a rationale in inclusion of hormonal measurements, especially cortisol, in both experimental and clinical studies of pain response [37, 38]. Estimation of cortisol levels remain a well proven and common tool for pain evaluation, and together with other physiological parameters, such as lipid profile and oxidative stress biomarkers, are strongly associated with the chronic pain [39–42]. Physiological markers have high sensitivity and specificity, and are regarded as indices of homeostatic changes that follow a painful event [43]. Thus, we focused on the endocrine, metabolic and redox parameters in the present work to monitor the changes in the adaptive biological mechanisms in response to chronic pain relative to the difference in the type of castration.

Castration by latex band, rubber ring, and Burdizzo in calves elicited greater cortisol response than that seen in the surgical procedure [44, 45]. Several studies indicated only a transient increase in cortisol level that rapidly returns to the baseline following the surgical castration [30, 32, 46]. This fluctuation in cortisol is followed by a rapid healing in superficial wounds in comparison to other androgen-eliminating practices as ring or Burdizzo castration and might cause less pain in the longer term [47]. One of the limitations of the present study was the measurement of cortisol levels only at the last time point.

Serum TC level in the S group was significantly lower than that observed in the Ch group at day 45 post-castration (77.70 ± 0.35 and 100.50 ± 1.28 mg/dl, respectively). Similarly, the *Longissimus thoracis* muscle in surgically castrated bulls contained lower cholesterol content than that found in the immunocastrated ones using luteinizing hormone-releasing hormone fusion protein vaccine [48]. The reduction in serum TC level in the S group most probably due to the decrease in the transcript level of hepatic 3-hydroxy-3-methylglutaryl-CoA reductase, a rate-limiting enzyme in cholesterol synthesis [49]. On the other hand, its activity and mRNA level were not changed by castration in pigs [50]. This implies the presence of species-specific variations in response to androgen deficiency due to differences in the expression of genes involved in metabolic pathways [9]. Castration augments the cellular uptake of cholesterol and fatty acids and suppresses lipolysis resulting in depletion of TC from circulation. For instance, gene expression of lipase G was up-regulated while that of phosphatidic acid phosphatase type 2B was down-regulated in the androgen-deprived pig model reflecting increased translocation of lipids from the extracellular space to the lipid storage sites and inhibition of lipolysis [8].

The obvious elevation in LDL-C at day 30 post-castration in the S group in comparison with the Ch group is similar to that previously found by Baik et al. [49] and Cai et al. [50] in cattle and pigs, respectively. Taken into consideration the fact that LDL receptors are responsible for the endocytosis of cholesterol-rich LDL thus, the decrease in its expression is associated with an increase in serum LDL level as that observed in surgically castrated pig model due to decreased removal of LDL-C from the blood stream [50]. It was found that increased expression of proprotein convertase subtilisin/kexin type 9, a protease enzyme, enhances the endosomal degradation of LDL receptors, and increases hepatic fat deposition by castration promoting down-regulation of LDL receptors that incriminates in the accumulation of LDL-C in the circulation [50]. *Longissimus thoracis* muscle of *Bos indicus* bulls subjected to the surgical castration contained lower amounts of polyunsaturated and monounsaturated fatty acids and higher amounts of saturated fatty acids relative to the immunocastrated ones [48]. Contents of polyunsaturated fatty acids in intramuscular and backfat of pigs were lower in pigs submitted to the surgical castration that those submitted to the immunocastration [51]. Given that polyunsaturated and monounsaturated fatty acids reduce LDL-C while saturated fatty acids cause the reverse outcome [52, 53], it was suggested that the raise in LDL-C at 30- day post-castration in the S group in comparison with Ch group might be attributed to specific-related modulations in fatty acid proportions relative to the types of castration. The increase in LDL-C following the surgical castration could increase the risk of atherosclerosis and other cardiovascular diseases [54].

The marked drop in HDL-C in the S group at day 30 and 60 post-castration is in consistent with that found in castrated rats and some epidemiological studies [55–57], in contrast to other studies demonstrating non-significant change [9] or elevation [8] in its level following the castration. TG enrichment of HDL particles, enhancement of lipolytic activity of hepatic lipase, and inhibition of hepatic production of apolipoprotein A-1 may contribute to the reduction in serum HDL-C [58, 59].

The changes in lipid profile which were observed following the surgical castration could have harmful consequences especially on the cardiovascular system. HDL-C encourages the reverse cholesterol transport, and its high levels are a prerequisite for the protection of vasculature from hyperlipidemia [60]. Therefore, its reduction together with the increase in LDL-C may give insight into the disturbance in pro-atherogenic/atherogenic lipoproteins balance which could be a contributory factor in the vascular defects. High level of TG and low level of HDL-C are implicated in the increased incidence of atherosclerosis and inflammatory reactions [61].

The significant elevation in TG levels at the last two-time points compared to the baseline following chemical loss of gonadal function could be secondary to the increased cortisol levels, as glucocorticoids stimulate the activity of enzymes involved in the hepatic TG synthesis [62].

The chemical castration in the current study caused a marked increase in LDL-C level at day 45 compared to the surgical castration (30.31 ±, 7.87 and 6.60 ± 0.12 mg/dl respectively). Glucocorticoids encourage VLDL secretion probably by stimulating the production and inhibiting the degradation of apolipoprotein B [63]. Therefore, the rate of VLDL production rates are elevated when the body is exposed to a high level of cortisol for a long period, and since VLDL clearance is unaltered this account for increased circulating VLDL and LDL levels [64].

The elevation in serum TPX of donkeys submitted to the surgical castration compared to those submitted to the chemical castration at day 45 is in parallel with the disturbance in the redox homeostasis of surgically castrated dogs [15]. The surgical castration in pigs encouraged lipid peroxidation in the pork sausage more than that found in the immunological one [65] due to the ability of the surgical castration to increase the polyunsaturated fatty acids, which subsequently enhances the susceptibility of cells to free radical attack [66, 67]. The ability of the current open method of castration to induce an obvious reduction in testosterone level could explain the increase in TPX level. This is highlighted by the induction of oxidative stress markers in several testosterone-deprived animal models through up-regulation of their gene expression, depletion of enzymatic and no-enzymatic antioxidants, and overproduction of reactive oxidants [68, 69]. The increase in TPX level denotes the excessive generation of reactive oxygenous and nitrogenous species under testosterone deprivation overcoming the antioxidant protective network and resulting in the initiation and propagation of chain reactions. This subsequently culminates in damaging vital macromolecules (including phospholipid, protein and DNA), triggering deleterious alterations on the levels of cellular membranes, enzymes, receptors, and genomic materials.

The chemical castration in our study fails to change TPX levels at all time points in comparison with day 0. Again, our findings are contradictory with the published research series by Jana and his collaborators who reported an obvious increase in testicular malondialdehyde and conjugated dienes content following intratesticular injection of CaCl_2_ solution indicating an increased rate of lipid peroxidation [3–7]. It must be taken into account that the previous studies focused on the oxidant/antioxidant balance in the testicular milieu, while our study dealt with that of the whole body. Moreover, the differential response of the androgenic testicular activity to CaCl_2_ injection in our earlier findings versus the previous research teamwork adds another explanation to the failure of chemical castration to modify the redox parameters in our investigation.

The decrease in TPX level at day 45 in the Ch group relative to the S group suggests the inverse relationship between cortisol level and lipid peroxidation. Prolonged administration of cortisol suppresses free radical, generation leading to decrease in lipid peroxidation products as the stress-realizing reactions activate stress-attenuating mechanisms including up-regulation of antioxidants [70–72].

When TPX level and TAC were compared at day 45 post-castration in both groups, it was found that serum TPX level increased while TAC decreased in the surgically castrated group in comparison with the chemically castrated one. At day 60, both of these parameters returned to the baseline in both groups. These outcomes reveal the ability of antioxidants to neutralize the reactive free radicals and restore the oxidant/antioxidant balance as some of the members of reactive oxidants act as signaling molecules to stimulate redox-sensitive transcription factors which up-regulate the gene expression of the protective antioxidant enzymes and proteins [73, 74]. TAC at the end of experimental period (day 14 post-castration) in surgically castrated dogs was lower than that observed at the previous time points (after recovery, and on days 3, 7 and 10 post-castration) denoting consumption of antioxidants in fighting the reactive oxidants [19]. However, the surgical procedure induced the antioxidant capacity of *Longissimus dorsi* muscle and backfat of pigs, estimated by 2, 2-diphenyl-1-picrylhydrazyl assay, in comparison with the immunocastration [75].

## Conclusion

Intratesticular injection of CaCl_2_ induced long-term stress response by activating HPA, releasing an excess amount of cortisol. The surgical castration is incriminated by evoking oxidative stress, which rapidly subsided 2 months after the surgical operation probably due to compensatory up-regulation in the antioxidant defense mechanisms. Both types of castration modified the main constituents of lipid profile. Further studies are highly recommended to highlight the changes in other physiological aspects related to the difference in the types of castration.

## Methods

### Animals

The present study was approved by the National Ethical Committee of The Faculty of Veterinary Medicine, Assiut University, Assiut, Egypt, that follows The OIE standards for the use of animals in research and education. The study was conducted on twelve clinically healthy adult male donkeys (*Equus asinus*), 2–3 years old and weighing 185–245 kg body weight (BW). Donkeys were obtained from the Experimental Animal House, Veterinary Teaching Hospital, Faculty of Veterinary Medicine, Assiut, Egypt, and kept in standard stable with ad libitum access to feed and water. Donkeys were allocated randomly and equally into two groups; the chemical (Ch) and surgical (S) groups (*n* = 6). Chemical castration was carried out under the effect of intravenous 2% xylazine HCl at a dose of 1 mg/kg BW (Xyla-Ject, ADWIA Co., SAE, Egypt) and local infiltration of the spermatic cord of each testicle with ten ml 2% lidocaine HCl (Dibucaine, Sigma-Tec Pharmaceutical Industry Co., Egypt) [76]. Donkeys in the Ch group received a single bilateral intratesticular injection of 20% calcium chloride dissolved in absolute ethanol in a dose of 20 ml/testis. A 21G × 1.5 needle was directed from the caudoventral aspect of each testis approximately 0.5 cm from the epididymal tail towards the dorsocranial aspect of the testis. The solution was carefully deposited along the entire route by linear infiltration while withdrawing the needle from the proximal to the distal end. Precautions were taken to prevent the seepage of solution from the injection site [1]. Donkeys in the S group were subjected to traditional open surgical castration according to Wilson et al. [77]. The surgical castration was performed under the effect of intravenous anaesthesia using 2% xylazine HCl at a dose 1.1 mg/kg BW (Xyla-Ject, ADWIA Co., SAE, Egypt) and 5% Ketamine HCl at a dose of 2.2 mg/kg BW (Ketamine, Sigma-tec Pharmaceutical Industries, SAE, Egypt). Ten ml of 2% lidocaine HCl (Dibucaine, Sigma-Tec Pharmaceutical Industry Co., Egypt) was injected directly into each spermatic cord and five ml subcutaneously along the anticipated incision site over each testicle to achieve local anesthesia [78]. The donkey was positioned in lateral recumbency. The down testicle was performed first. Each testicle was exteriorized with its spermatic cord by a direct incision through the skin and the tunics. The cord was then emasculated and the skin incision was left to heal by second intention. Donkeys were recovered from anesthesia while positioned in lateral recumbency in a padded recovery stall, and were monitored until they were standing.

Donkeys of the Ch and S groups were injected intramuscularly with procaine penicillin and dihydrostreptomycin sulphate at a dose of 1 ml/kg BW (Pen & Strep, 1 ml contains procaine penicillin 200 mg and dihydrostreptomycin sulphate 250, Norbrook Laboratories Limited, Newry, BT35 6JP) and intravenously with phenylbutazone at a dose of 1.1 ml/50 kg BW (Phenyl-D 20%, 1 ml contains phenylbutazone 200 mg, DELTA PHARMA, veterinary sector, Egypt) for three successive days for microbial prophylaxis and pain management, respectively after each intervention.

Donkeys in both groups were kept under observation for 60 days. The animals were allowed to recover before being euthanised. At the end of the study, donkeys were sedated by intravenous injection of 2% xylazine hydrochloride (Xyla-Ject, ADWIA Co., SAE, Egypt) at a dose of 1.1 mg/kg BW followed by rapid intravenous injection of thiopental sodium (Thiopental sodium 1 g vial, EPICO, Egypt) at a dose of 35 mg/kg BW [77].

### Collection of blood samples

Blood samples were collected from the jugular vein under complete aseptic condition at 0830 a.m. at five-time points; prior to castration and at 15, 30, 45, and 60 days after the beginning of experiment. Serum was obtained by centrifugation at 3000 rpm for 10 min and stored at − 20 °C for estimation of biochemical parameters later on.

### Biochemical measurements

Serum cortisol level was assessed at the end of the experimental period as a chronic discomfort indicator using enzyme immunoassay test kit according to manufacturer^’^s instruction (Immunospec Corporation, Canoga Park, CA, USA) with a minimum detectable concentration of 0.366 μg/dl. Total cholesterol (TC), high density lipoprotein-cholesterol (HDL-C), and triglycerides (TG) levels were measured according to the manufacturer’s instructions using commercially available kits (Egyptian Company for Biotechnology, Egypt). Low density lipoprotein-cholesterol (LDL-C) was calculated according to Friedewald et al. [79]. Total peroxide (TPX) level was estimated as described elsewhere [80] and calculated from a curve constructed of serial standard concentrations. Following the reaction with a known amount of exogenous hydrogen peroxide, determination of the residual amount of antioxidants was used to reflect the total antioxidant capacity (TAC) of the sample [81]. Serum TC, HDL-C, TG, and TAC levels were measured colorimetrically using spectrophotometer (Spectronic 21, Moton Roy Company, USA). The oxidative stress index (OSI), as a biomarker of the overall redox potential of serum, was calculated as the percent ratio of TPX content to TAC concentration according to the following equation: OSI = TPX (μM/L) / TAC (μM/L) × 100 [80].

### Statistical analysis

The analysis was performed using GraphPad Prism 5 version 5.01 (GraphPad Software Inc., San Diego, CA, USA). The data were represented as means ± standard error of the mean (SEM). Repeated measures ANOVA was used to determine the effects of each type of castration as an independent variable on the levels of serum oxidative stress parameters and lipid profile as response variables followed by Tukey’s multiple comparison test. Independent-samples t-test was used to compare between the effects of each type of castration on serum cortisol level as a response variable at the last time point, and the rest of the measured parameters as response variables at each post-castration time point. Differences were considered statistically significant at *P* < 0.05.

## Data Availability

From the corresponding author.

## Abbreviations

CaCl_2_: Calcium chloride
TC: Total cholesterol
HDL-C: High density lipoprotein-cholesterol
TG: Triglycerides
LDL-C: Low density lipoprotein-cholesterol
TPX: Total peroxide
TAC: Total antioxidant capacity
OSI: Oxidative stress index
HPA: Hypothalamic-pituitary-adrenal axis

## References

[1] Ibrahim A, Ali MM, Abou-Khalil NS, Ali MF (2016). Evaluation of chemical castration with calcium chloride versus surgical castration in donkeys: testosterone as an endpoint marker. BMC Vet Res.

[2] Leoci R, Aiudi G, Cicirelli V, Brent L, Iaria C, Lacalandra GM (2019). Effects of intratesticular vs intraepididymal calcium chloride sterilant on testicular morphology and fertility in dogs. Theriogenology.

[3] Jana K, Samanta PK (2007). Sterilization of male stray dogs with a single intratesticular injection of calcium chloride: a dose-dependent study. Contraception.

[4] Jana K, Samanta PK (2006). Evaluation of single intratesticular injection of calcium chloride for nonsurgical sterilization in adult albino rats. Contraception.

[5] Jana K, Samanta PK, Ghosh D (2005). Evaluation of single intratesticular injection of calcium chloride for nonsurgical sterilization of male black Bengal goats (Capra hircus): a dose-dependent study. Anim Reprod Sci.

[6] Jana K, Samanta PK, Ghosh D (2002). Dose-dependent response to an intratesticular injection of calcium chloride for induction of chemosterilization in adult albino rats. Vet Res Commun.

[7] Jana K, Samanta PK (2011). Clinical evaluation of non-surgical sterilization of male cats with single intratesticular injection of calcium chloride. BMC Vet Res.

[8] Yao Y, Ma H, Wu K, Shao Y, Han W, Cai Z, Xu N, Qi M, Zhao C, Wu C (2018). Body composition, serum lipid levels, and transcriptomic characterization in the adipose tissue of male pigs in response to sex hormone deficiency. Gene.

[9] Zhang Y, Wang H, Wang Y, Wang H, Zhang S, Hong J, Guo H, Chen D, Yang Y, Zan L (2017). Transcriptome analysis of mRNA and microRNAs in intramuscular fat tissues of castrated and intact male Chinese Qinchuan cattle. PLoS One.

[10] Christoffersen B, Raun K, Svendsen O, Fledelius C, Golozoubova V (2006). Evalution of the castrated male Sprague–Dawley rat as a model of the metabolic syndrome and type 2 diabetes. Int J Obes.

[11] Yang Q, Vijayakumar A, Kahn BB (2018). Metabolites as regulators of insulin sensitivity and metabolism. Nat Rev Mol Cell Biol.

[12] Taylor LE, Gillis EE, Musall JB, Baban B, Sullivan JC (2018). High-fat diet-induced hypertension is associated with a proinflammatory T cell profile in male and female Dahl salt-sensitive rats. Am J Phys Heart Circ Phys.

[13] Gulhane M, Murray L, Lourie R, Tong H, Sheng YH, Wang R, Kang A, Schreiber V, Wong KY, Magor G (2016). High fat diets induce colonic epithelial cell stress and inflammation that is reversed by IL-22. Sci Rep.

[14] Dunn SM, Hilgers RHP, Das KC (2017). Decreased EDHF-mediated relaxation is a major mechanism in endothelial dysfunction in resistance arteries in aged mice on prolonged high-fat sucrose diet. Phys Rep.

[15] Mogheiseh A, Koohi F, Nazifi S, Tabrizi AS, Taheri P, Salavati S (2019). Oxidative-antioxidative status and hepatic and renal factors following melatonin administration in castrated and intact dogs. Basic Clin Androl.

[16] Rahal A, Kumar A, Singh V, Yadav B, Tiwari R, Chakraborty S, Dhama K (2014). Oxidative stress, prooxidants, and antioxidants: the interplay. Biomed Res Int.

[17] Durand D (2019). Faure M, de la Foye a, des Roches ADB: benefits of a multimodal analgesia compared to local anesthesia alone to alleviate pain following castration in sheep: a multiparametric approach. Animal.

[18] Mellor DJ, Stafford KJ (2000). Acute castration and/or tailing distress and its alleviation in lambs. N Z Vet J.

[19] Aengwanich W, Sakundech K, Chompoosan C, Tuchpramuk P, Boonsorn T (2019). Physiological changes, pain stress, oxidative stress, and total antioxidant capacity before, during, and after castration in male dogs. J Vet Behav.

[20] Gao ST, Lu M, Zhou Z, Zhou ZK, Baumgard LH, Jiang D, Bionaz M, Bu DP (2019). Heat stress negatively affects the transcriptome related to overall metabolism and milk protein synthesis in mammary tissue of midlactating dairy cows. Physiol Genomics.

[21] Solin AV, Korozin VI, Lyashev YD (2013). Effects of regulatory peptides on the stress-induced changes of lipid metabolism in experimental animals. Bull Exp Biol Med.

[22] Marik PE (2006). Dyslipidemia in the critically ill. Crit Care Clin.

[23] Peckett AJ, Wright DC, Riddell MC (2011). The effects of glucocorticoids on adipose tissue lipid metabolism. Metabolism.

[24] Cole C, Bentz B, Maxwell L (2014). Equine pharmacology: Jones Wiley & Sons.

[25] Oliveira FC, Ferreira CER, Haas CS, Oliveira LG, Mondadori RG, Schneider A, Rovani MT, Gonçalves PBD, Vieira AD, Gasperin BG (2017). Chemical castration in cattle with intratesticular injection of sodium chloride: effects on stress and inflammatory markers. Theriogenology.

[26] Martins LT, Gonçalves MC, Tavares KCS, Gaudêncio S, Neto PCS, Dias A, Gava A, Saito M, Oliveira C, Mezzalira A, Vierira AD (2011). Castration methods do not affect weight gain and have diverse impacts on the welfare of water buffalo males. Livest Sci.

[27] Carroll JA, Berg EL, Strauch TA, Roberts MP, Kattesh HG (2006). Hormonal profiles, behavioral responses, and short-term growth performance after castration of pigs at three, six, nine, or twelve days of age. J Anim Sci.

[28] Li S, Nitsos I, Polglase GR, Newnham JP, Challis JRG, Moss TJM (2013). Effects of tail docking and castration on stress responses in lambs and the influence of prenatal glucocorticoid treatment. Reprod Fertil Dev.

[29] Davis JR, Langford GA, Kirby PJ, Johnson WR, Gomes WR (1970). The testicular capsule. The testis.

[30] Webster HB, Morin D, Jarrell V, Shipley C, Brown L, Green A, Wallace R, Constable PD (2013). Effects of local anesthesia and flunixin meglumine on the acute cortisol response, behavior, and performance of young dairy calves undergoing surgical castration. J Dairy Sci.

[31] Yoo SP, Baik M, Kang HJ, Park SJ, Seok-Hyeon B, Jeong I (2019). Effects of castration stress on behaviors and leukocyte cytokine gene expression in Korean cattle bull calves. J Anim Sci.

[32] Laurence M, Barnes A, Collins T, Hyndman T, Musk GC (2018). Assessing and mitigating post-operative castration pain in Bos indicus cattle. Anim Prod Sci.

[33] Dalla Costa E, Minero M, Lebelt D, Stucke D, Canali E, Leach MC (2014). Development of the horse grimace scale (HGS) as a pain assessment tool in horses undergoing routine castration. PLoS One.

[34] Love EJ, Taylor PM, Clark C, Whay HR, Murrell J (2009). Analgesic effect of butorphanol in ponies following castration. Equine Vet J.

[35] Ashley FH, Waterman-Pearson AE, Whay HR (2005). Behavioural assessment of pain in horses and donkeys: application to clinical practice and future studies. Equine Vet J.

[36] Gleerup KB, Forkman B, Lindegaard C, Andersen PH (2015). An equine pain face. Vet Anaesth Analg.

[37] Virgin J, Hendrickson D, Wallis T, Rao S (2010). Comparison of intraoperative behavioral and hormonal responses to noxious stimuli between mares sedated with caudal epidural detomidine hydrochloride or a continuous intravenous infusion of detomidine hydrochloride for standing laparoscopic ovariectomy. Vet Surg.

[38] Erber R, Wulf M, Becker-Birck M, Kaps S, Aurich JE, Möstl E, Aurich C (2012). Physiological and behavioural responses of young horses to hot iron branding and microchip implantation. Vet J.

[39] Landa L (2012). Pain in domestic animals and how to assess it: a review. Vet Med.

[40] Lawson AL, Opie RR, Stevens KB, Knowles EJ, Mair TS (2019). Application of an equine composite pain scale and its association with plasma adrenocorticotropic hormone concentrations and serum cortisol concentrations in horses with colic. Equine Vet Educ.

[41] Cowen R, Stasiowska MK, Laycock H, Bantel C (2015). Assessing pain objectively: the use of physiological markers. Anaesthesia.

[42] Herzberg D, Strobel P, Chihuailaf R, Ramirez-Reveco A, Müller H, Werner M, Bustamante H (2019). Spinal reactive oxygen species and oxidative damage mediate chronic pain in lame dairy cows. Animals.

[43] McGrath PJ, Stevens BJ, Walker SM, Zempsky WT (2013). Oxford textbook of paediatric pain: OUP Oxford.

[44] Stafford KJ, Mellor DJ, Todd SE, Bruce RA, Ward RN (2002). Effects of local anaesthesia or local anaesthesia plus a non-steroidal anti-inflammatory drug on the acute cortisol response of calves to five different methods of castration. Res Vet Sci.

[45] Robertson IS, Kent JE, Molony V (1994). Effect of different methods of castration on behaviour and plasma cortisol in calves of three ages. Res Vet Sci.

[46] Sutherland MA, Davis BL, Brooks TA, McGlone JJ (2010). Physiology and behavior of pigs before and after castration: effects of two topical anesthetics. Animal.

[47] Stafford KJ, Mellor DJ (2005). The welfare significance of the castration of cattle: a review. N Z Vet J.

[48] Ruiz MR, Matsushita M, Visentainer JV, Hernandez JA, Ribeiro EL, Shimokomaki M, Reeves JJ, De Souza NE (2005). Proximate chemical composition and fatty acid profiles of Longissimus thoracis from pasture fed LHRH immunocastrated, castrated and intact Bos indicus bulls. S Afr J Anim Sci.

[49] Baik M, Nguyen TH, Jeong JY, Piao MY, Kang HJ (2015). Effects of castration on expression of lipid metabolism genes in the liver of korean cattle. Asian Australas J Anim Sci.

[50] Cai Z, Xi H, Pan Y, Jiang X, Chen L, Cai Y, Zhu K, Chen C, Xu X, Chen M (2015). Effect of testosterone deficiency on cholesterol metabolism in pigs fed a high-fat and high-cholesterol diet. Lipids Health Dis.

[51] Grela ER, Kowalczuk-Vasilev E, Klebaniuk R (2013). Performance, pork quality and fatty acid composition of entire males, surgically castrated or immunocastrated males, and female pigs reared under organic system. Pol J Vet Sci.

[52] Bonanome A, Pagnan A, Biffanti S, Opportuno A, Sorgato F, Dorella M, Maiorino M, Ursini F (1992). Effect of dietary monounsaturated and polyunsaturated fatty acids on the susceptibility of plasma low density lipoproteins to oxidative modification. Arterioscler Thromb.

[53] Jansen S, López-Miranda J, Castro P, López-Segura F, Marín C, Ordovás JM, Paz E, Jiménez-Perepérez J, Fuentes F, Pérez-Jiménez F (2000). Low-fat and high-monounsaturated fatty acid diets decrease plasma cholesterol ester transfer protein concentrations in young, healthy, normolipemic men. Am J Clin Nutr.

[54] Wadhera RK, Steen DL, Khan I, Giugliano RP, Foody JM (2016). A review of low-density lipoprotein cholesterol, treatment strategies, and its impact on cardiovascular disease morbidity and mortality. J Clin Lipidol.

[55] Mäkinen JI, Perheentupa A, Irjala K, Pöllänen P, Mäkinen J, Huhtaniemi I, Raitakari OT (2008). Endogenous testosterone and serum lipids in middle-aged men. Atherosclerosis.

[56] Olayaki LA, Alagbonsi AI, Adamson M, Ayodele OD, Oyewopo AO (2016). Vitamin C alleviates surgical castration-induced dyslipidemia in male rats. Niger J Cardiol.

[57] Akishita M, Fukai S, Hashimoto M, Kameyama Y, Nomura K, Nakamura T, Ogawa S, Iijima K, Eto M, Ouchi Y (2010). Association of low testosterone with metabolic syndrome and its components in middle-aged Japanese men. Hypertens Res.

[58] Rashid S, Watanabe T, Sakaue T, Lewis GF (2003). Mechanisms of HDL lowering in insulin resistant, hypertriglyceridemic states: the combined effect of HDL triglyceride enrichment and elevated hepatic lipase activity. Clin Biochem.

[59] Zmuda JM, Cauley JA, Kriska A, Glynn NW, Gutai JP, Kuller LH (1997). Longitudinal relation between endogenous testosterone and cardiovascular disease risk factors in middle-aged men: a 13-year follow-up of former multiple risk factor intervention trial participants. Am J Epidemiol.

[60] Kivelä AM, Dijkstra MH, Heinonen SE, Gurzeler E, Jauhiainen S, Levonen A, Ylä-Herttuala S (2012). Regulation of endothelial lipase and systemic HDL cholesterol levels by SREBPs and VEGF-A. Atherosclerosis.

[61] Welty FK (2013). How do elevated triglycerides and low HDL-cholesterol affect inflammation and atherothrombosis?. Curr Cardiol Rep.

[62] Pittner RA, Fears R, Brindley DN (1985). Interactions of insulin, glucagon and dexamethasone in controlling the activity of glycerol phosphate acyltransferase and the activity and subcellular distribution of phosphatidate phosphohydrolase in cultured rat hepatocytes. Biochem J.

[63] Wang Y, Jones Voy B, Urs S, Kim S, Soltani-Bejnood M, Quigley N, Heo Y, Standridge M, Andersen B, Dhar M (2004). The human fatty acid synthase gene and de novo lipogenesis are coordinately regulated in human adipose tissue. J Nutr.

[64] Taskinen MR, Nikkila EA, Pelkonen R, Sane T (1983). Plasma lipoproteins, lipolytic enzymes, and very low density lipoprotein triglyceride turnover in Cushing’s syndrome. J Clin Endocrinol Metab.

[65] Jones-Hamlow KA, Tavárez MA, Schroeder AL, Dilger AC (2016). Lipid oxidation, sensory characteristics, and color of fresh pork sausage from immunologically castrated pigs stored frozen for up to 12 weeks. Food Sci Nutr.

[66] Asmus MD, Tavarez MA, Tokach MD, Dritz SS, Schroeder AL, Nelssen JL, Goodband RD, DeRouchey JM (2014). The effects of immunological castration and corn dried distillers grains with solubles withdrawal on growth performance, carcass characteristics, fatty acid analysis, and iodine value of pork fat depots. J Anim Sci.

[67] Di Nunzio M, Valli V, Bordoni A (2016). PUFA and oxidative stress. Differential modulation of the cell response by DHA. Int J Food Sci Nutr.

[68] Kataoka T, Hotta Y, Maeda Y, Kimura K (2017). Testosterone deficiency causes endothelial dysfunction via elevation of asymmetric dimethylarginine and oxidative stress in castrated rats. J Sex Med.

[69] Shiota M, Yokomizo A, Naito S (2011). Oxidative stress and androgen receptor signaling in the development and progression of castration-resistant prostate cancer. Free Radic Biol Med.

[70] Flerov MA, Gerasimova IA, Rakitskaya VV (2003). Lipid peroxidation in the striatum of rats during stress after administration of cortisol. Neurosci Behav Physiol.

[71] Atanasova S, Wieland E, Schlumbohm C, Korecka M, Shaw L, von Ahsen N, Fuchs E, Oellerich M, Armstrong V (2009). Prenatal dexamethasone exposure in the common marmoset monkey enhances gene expression of antioxidant enzymes in the aorta of adult offspring. Stress.

[72] Yoshioka T, Kawamura T, Meyrick BO, Beckman JK, Hoover RL, Yoshida H, Ichikawa I (1994). Induction of manganese superoxide dismutase by glucocorticoids in glomerular cells. Kidney Int.

[73] Rahman I (2000). Regulation of nuclear factor-κB, activator protein-1, and glutathione levels by tumor necrosis factor-α and dexamethasone in alveolar epithelial cells. Biochem Pharmacol.

[74] Giles GI, Jacob C, Winyard PG (2009). Redox-controlled transcription factors and gene expression. Redox signaling and regulation in biology and medicine.

[75] Ježek F, Abdullah FAA, Steinhauserová I, Hulánková R, Bořilová G (2019). Comparison of oxidation status and antioxidant capacity of meat from surgically castrated and immunocastrated pigs, entire males and sows. Acta Vet Brno.

[76] Pereira LF, Dias FGG, Miguel MP, Honsho CS, Tavares DC, Hellú JAA, Souza FF (2018). Testicular histological evaluation and serum testosterone concentrations of bulls after chemical castration with calcium chloride. Pesqui Vet Bras.

[77] Wilson DA, Kramer J, Constantinescu GM, Branson KR (2008). Manual of equine field surgery, p 186–190.

[78] Kilcoyne I (2013). Equine castration: a review of techniques, complications and their management. Equine Vet Educ.

[79] Friedewald WT, Levy RI, Fredrickson DS (1972). Estimation of the concentration of low-density lipoprotein cholesterol in plasma, without use of the preparative ultracentrifuge. Clin Chem.

[80] Harma M, Harma M, Erel O (2005). Measurement of the total antioxidant response in preeclampsia with a novel automated method. Eur J Obstet Gynecol Reprod Biol.

[81] Koracevic D, Koracevic G, Djordjevic V, Andrejevic S, Cosic V (2001). Method for the measurement of antioxidant activity in human fluids. J Clin Pathol.

